# Examining the Pathoplastic Moderating Role of Education on the Association between Depressive Mood and Self-Rated Health among Cancer Survivors: A Population-Based Study

**DOI:** 10.3390/curroncol28050343

**Published:** 2021-10-11

**Authors:** Anao Zhang, Kaipeng Wang, Adam S. DuVall

**Affiliations:** 1 School of Social Work, University of Michigan, Ann Arbor, MI 48109, USA; 2 Adolescent and Young Adult Oncology Program, Michigan Medicine, Ann Arbor, MI 48109, USA; 3 Graduate School of Social Work, University of Denver, Denver, CO 80208, USA; kaipeng.wang@du.edu; 4 Department of Medicine, University of Chicago, Chicago, IL 60637, USA; duvalla@medicine.bsd.uchicago.edu

**Keywords:** pathoplasticity, education, depression, self-rated health, cancer survivor

## Abstract

Objective: Self-rated health (SRH) is a salient patient outcome for cancer survivors, and depressive mood and education are known determinants of cancer survivors’ SRH. Moving beyond the well-established direct association between depressive mood, education, and SRH among cancer survivors, this epidemiological study investigated the pathoplastic role of education on depressive mood in relation to SRH among a nationally representative sample of cancer survivors in the United States. Methods: The 2019 National Health Interview Survey was analyzed using data from adult participants (≥18 years old) who self-reported as cancer survivors (*n* = 3844). Ordered logistic regression was used to evaluate the direct impact of depressive mood and education in relation to SRH. In addition, the pathoplastic moderating effect was evaluated using ordered logistic regression with an interaction term of depressive mood and education in the regression model. All analyses adjusted for complex sample weights so that findings are nationally representative. Results: After adjusting for all covariates, U.S. cancer survivors’ depressive mood was significantly associated with lower SRH, and U.S. cancer survivors’ higher education was significantly associated with higher SRH. As a pathoplastic moderator, cancer survivors’ education significantly moderated the association between depressive mood and SRH. The negative association between depressive mood and SRH was significantly greater among those with higher education. Conclusion: Moving beyond the direct association between depressive mood, education, and SRH, education served as a pathoplastic moderator in relation to depressive mood and SRH. Psycho-oncology providers need to be mindful of the “protective-risk” effect of education in relation to cancer survivors’ depressive mood and SRH.

## 1. Introduction

Self-rated health (SRH) is a salient general health status indicator for cancer survivors because it indicates a set of robust clinical outcomes, including cancer patients’ ability to manage and cope with treatment-related symptoms, quality of life, and mortality [1,2,3]. SRH is a significant patient-reported outcome measure (PROM) among cancer patients, which has the strongest predictive validity for health status when compared with any other single-item health rating question, such as physicians’ assessment or BMI [1,4,5]. Therefore, it is essential for oncology providers to shift from a physician-centered approach to patient-centered medicine, especially when promoting the general health status of individuals diagnosed with cancer during and after their treatment [6,7,8]. A compelling body of social determinants of health literature has identified that demographic, e.g., race and socioeconomic status, and psychosocial factors, e.g., depression and social support, are significant predictors of SRH among cancer survivors [9,10,11]. Depression, a prevalent mental health condition among cancer survivors, is negatively associated with SRH [12,13]. One emerging theory explicitly linking depression and low SRH is psychoneuroimmunology [14], which articulates that depressed mood triggers chronic stress and, through neuroendocrine pathways, impacts individuals’ immune system so that individuals are likely to report low SRH as depression worsens [15]. Education, one of the key variables representing individual socioeconomic status, is another established factor associated with cancer survivors’ SRH [2,11]. Individuals with higher levels of education are expected to have better access to healthcare resources and are likely to report a higher level of SRH [16,17].

Despite the well-studied direct association between depression, education, and cancer survivors’ SRH, few studies have evaluated the dynamic interaction between depression and education concerning cancer survivors’ SRH, also known as a pathoplastic relationship [18]. Pathoplasticity is a theoretical framework that emphasizes the role of an individual’s acquired personality (e.g., temperament, interpersonal style, and cultural influence) in the manifestation of psychological disorders, such as onset, chronicity, and symptom severity [18,19]. Educational attainment has been associated with different expressions of depressive disorders [20,21,22]. For example, Di Florio and colleagues [20] found that higher education is positively associated with more severe expressions of depression, e.g., feelings of being scared or panicky and suicidal ideation, whereas those with low education are more likely to report a different symptom profile, such as anhedonia and guilty feelings. Similarly, Gan and colleagues [21] found that more years of education is associated with apathy and hypersomnia, as well as a greater chance of recurrent major depressive disorder, suggesting that educational attainment may not be protective, but instead a risk factor, for individuals with depression.

When available theoretical and empirical evidence is taken together, the pathoplastic relationship between depression and education underscores the necessity to evaluate further their impact in relation to SRH on cancer survivors. It is essential to evaluate beyond the direct association between depression, education, and SRH and explore the possible pathoplastic moderating role of education on the association between depression and SRH in cancer survivors. Thus, in addition to the positive direct association between education and cancer survivors’ SRH, evaluating the potential influence of education on the relationship between depression and SRH among cancer survivors is warranted. Using a national representative dataset, the National Health Interview Survey, this study aims to test the following hypotheses: (1) cancer survivors’ depressive mood is negatively associated with SRH; (2) cancer survivors’ education is positively associated with SRH; and (3) to explore whether and how education moderates the association between depressive mood and cancer survivors’ SRH.

## 2. Materials and Methods

### 2.1. Data Source

This study used the 2019 National Health Interview Survey (NHIS), a national representative cross-sectional household survey on a broad range of health topics from the non-institutionalized civilian adult population of the United States [23]. The NHIS uses clustered sampling techniques to maximize its representation of the U.S. population and is conducted in a face-to-face format. The sampling process of NHIS started with partitioning the United States into 1689 geographic areas, with each area representing one stratum or two strata, depending on the state. A cluster of addresses was then defined within each stratum, and approximately 2500 addresses were included in each cluster. Within each stratum, a specific number of clusters was systematically chosen proportional to the number of clusters in the strata, i.e., a larger stratum has more clusters selected, resulting in a nationally representative sample. For this study, we selected U.S. adults (18 years or older) who self-identified as cancer survivors by responding “yes” to the question “Have you ever been told by a doctor or other health professional that you had cancer?” A total number of 3844 participants were included in the analytical sample. Institutional Review Board (IRB) approval was exempted because the study used publicly available de-identified data.

### 2.2. Measures

#### 2.2.1. Self-Rated Health (SRH)

SRH was measured by asking participants, “Would you say your health, in general, is excellent, very good, good, fair, or poor?” Participants responded to a 5-point Likert scale, and responses were coded so that higher score represents better SRH: 1 = poor, 2 = fair, 3 = good, 4 = very good, and 5 = excellent.

#### 2.2.2. Depressive Mood

Depressive mood was measured by the 8-item Patient Health Questionnaire (PHQ-8) [24]. Participants were asked to respond to 8 screening questions and indicate how often they had been bothered by these 8 questions over the past 2 weeks. The PHQ-8 consists of 8 of the 9 criteria on which the DSM-5 diagnosis of depressive disorders is based [25], excluding the question on suicidal or self-injurious thoughts. Some PHQ-8 example inquiries are: (1) little interest or pleasure in doing things; (2) feeling down, depressed, or hopeless; and (3) trouble concentrating on things, such as reading the newspaper or watching television. Participants responded to a 4-point Likert scale: 1 = not at all, 2 = several days, 3 = more than half the days, and 4 = nearly every day. A summed score of responses to all eight items, ranging from 8 to 32, was calculated to reflect the severity of depressive mood, with a higher score indicating greater severity of depressive mood (Cronbach’s α = 0.84).

#### 2.2.3. Education

Participants’ educational attainment was measured by asking them about their highest level of education received. Participants chose from one of the following categories: (1) below high school (reference group), (2) high school or equivalent, (3) some college, (4) bachelor’s degree, and (5) above bachelor’s degree, and were coded as dummy variables for data analysis.

#### 2.2.4. Covariates

Controlled covariates included age group (1 = young adults, i.e., 18–39 years old (reference group); 2 = middle adults, i.e., 40–64 years old; and 3 = older adults, i.e., 65 years or older), sex (0 = male, 1 = female), race (dummy coded for Non-Hispanic White (reference group), Hispanic, Non-Hispanic Black/African American only, Non-Hispanic Asian only, Non-Hispanic American Indian or Alaska Native only, Non-Hispanic American Indian or Alaska Native and other races, and other single and multiple races), income (measured by percentage of the Federal Poverty Line (FPL), dummy coded for below 100% FPL (reference), 100% to below 200% FPL, 200% to below 300% FPL, 300% to below 400% FPL, 400% to below 500% FPL, and 500% FPL and above), marital status (dummy coded for married (reference group), separated, divorced, single/never married, and widowed), residential area (dummy coded for large central metro area (reference group), large fringe metro area, medium and small metro area, and non-metropolitan area), chronic health conditions other than cancer (including hypertension, high cholesterol, asthma, diabetes, COPD, arthritis, and coronary heart disease (for each condition 0 = no, 1 = yes)), and a diagnosis of at least one of 29 cancer diagnoses from doctors or other health professionals (0 = no, 1 = yes for each cancer diagnosis).

### 2.3. Statistical Analysis

Participant characteristics were summarized using descriptive statistics. Two ordered logistic regression models were estimated to (1) evaluate the association between depressive mood, education, and SRH among cancer survivors (in Model 1) and (2) examine the moderation effect of education on the association between depressive mood and SRH by introducing an interaction term: depressive mood × education (in Model 2). Both Model 1 and 2 analyses controlled for all covariates. Post hoc analyses based on Model 2 results were conducted to investigate the association between depressive mood and SRH for cancer survivors, with each level of education adjusting for all covariates. Specifically, we estimated the conditional marginal effect of depressive mood on SRH, i.e., the odds ratio of reporting a higher rank of SRH for depressive mood, by education after controlling for all covariates.

An important assumption in ordered logistic regression is that the effect of any independent variables should be consistent or proportional across the different thresholds of the outcome, which can be globally tested by the Brant test [26]. Rejection of the test indicates a violation of the proportional odds assumption. In this study, using the original 5-category outcome measure for SRH led to a violation of the proportionality of odds assumption, $\chi^{2}$(173) = 251.16, *p* < 0.001 for Model 1 and $\chi^{2}$(185) = 252.55, *p* < 0.001 for Model 2. In order to address the proportionality of odds assumption violation while maintaining the variability of the SRH outcome, “very good” and “excellent” SRH were combined into one category, i.e., 1 = poor; 2 = fair; 3 = good; 4 = very good/excellent. This way of coding SRH did not violate the proportionality of odds assumption, $\chi^{2}$(118) = 133.08, *p* = 0.162 for Model 1 and $\chi^{2}$(126) = 131.38, *p* = 0.353 for Model 2. Although missing values account for only 2.1% or less of all variables, it is important to assess whether the missing pattern is completely random (MCAR) and thus determine whether ignoring the cases with missing values will bias the results. The purpose of Little’s MCAR test is to determine whether the difference in the means of different missing-value patterns was statistically significant [27]. Rejection of the test shows missing values are not completely at random, which indicates simply ignoring cases with missing values will bias the results. In this study, Little’s MCAR test, $\chi^{2}$(414) = 569.08, *p* < 0.001, rejected the assumption of missing completely at random. Therefore, multiple imputations by chained equations were used to address potential biases caused by missing values [28,29]. All model parameters were estimated based on pooled results from 20 imputed datasets using Stata 15 SE [30]. All the parameters for ordered logistic regression models adjusted for sample weights to be representative of cancer survivors in the United States.

## 3. Results

### 3.1. Participant Characteristics

Table 1 presents descriptive statistics of participants’ characteristics. Over 40% of cancer survivors reported very good/excellent SRH, and about 32% of the cancer survivors reported good SRH, leaving 18.51% and 8.59% of the cancer survivors reporting fair and poor SRH, respectively. Participants’ PHQ-8 depressive mood score averaged at 11.02 (SD = 4.34), suggesting an average of moderate depressive mood severity among cancer survivors. The percentages of young adult, middle adult, and older adult cancer survivors were 4.6%, 31.14%, and 64.26%, respectively. Over half of the cancer survivors were female (58.43%), and about one-third had below high school (8.56%) or high school or equivalent education (24.25%), one-third had some college experience (30.41%), and about one-third had a bachelor’s degree (21.37%) or higher (15.4%). Most participants were Non-Hispanic White (86.08%), 5.05% of the participants were Hispanic, 5.52% of the participants were Non-Hispanic Black/African American only, and 1.22% and 0.57% of the participants were Non-Hispanic Asian only and Non-Hispanic American Indian/Alaska Native only, respectively. Half of the cancer survivors were married (49.55%), 1.04% were separated, 18.85% were divorced, 8.96% were single/never married, and 21.61% were widowed. The percentages of living in a large central metro area, large fringe metro area, medium and small metro area, and non-metropolitan area were 22.71%, 24.74%, 33.84%, and 18.70%, respectively. Income of below 100% Federal Poverty Line (FPL), 100%–below 200% FPL, 200%–below 300% FPL, 300%–below 400% FPL, 400%–below 500% FPL, and 500% FPL and above, respectively, accounted for 8.74%, 18.05%, 17.27%, 13.27%, 10.85%, and 31.82% of the sample. About half of the cancer survivors reported having hypertension (55.87%), high cholesterol (46.87%), and arthritis (46.72%). Over one-tenth of the participants reported having asthma (10.61%), diabetes (15.58%), COPD, emphysema, or chronic bronchitis (11.65%), and coronary heart disease (11.7%), respectively.

### 3.2. The Direct Association between Depressive Mood, Education, and SRH in Cancer Survivors

Table 2 Model 1 displays the results from the ordered logistic regression models evaluating the direct association between depressive mood, education, and SRH in cancer survivors. After adjusting for all covariates, U.S. cancer survivors’ depressive mood was found to be significantly associated with lower SRH, OR = 0.863, 95% CI: 0.840–0.886. In addition, U.S. cancer survivors’ educational attainment is significantly associated with their SRH. Compared to those with education of below high school, cancer survivors with education of high school or equivalent (OR = 1.466, 95% CI: 1.08–2.015), some college (OR = 1.762, 95% CI: 1.302–2.384), bachelor’s degree (OR = 2.358, 95% CI: 1.692–3.285), and above bachelor’s degree (OR = 2.039, 95% CI: 1.440–2.888) are significantly associated with higher SRH accounting for all covariates.

### 3.3. The Pathoplastic Moderating Effect of Education on the Association between Depressive Mood and SRH in Cancer Survivors

Table 2 Model 2 demonstrates the results with the interaction between depressive mood and education, testing the pathoplastic moderating effect of education. The results of the joint tests of all interaction term coefficients are statistically significant, *F*(3552.9) = 3.07, *p* < 0.05, indicating that the association between depressive mood and SRH differs by education. Specifically, the negative association between depressive mood and SRH in cancer survivors is significantly stronger for those with a bachelor’s degree (OR = 0.900, 95% CI: 0.837–0.967) and those with an above bachelor’s degree (OR = 0.859, 95% CI: 0.785–0.941) in comparison to those with an education level below high school.

Based on the results in Model 2, Table 3 displays the post-estimation findings of the association between depressive mood and SRH by education. The results indicate that after adjusting for all covariates, depressive mood is significantly associated with lower SRH for cancer survivors across all levels of education. However, such negative association, in general, became stronger with an increase in participants’ education levels, except for cancer survivors with some college experience.

## 4. Discussion

To our knowledge, this study is among the first to investigate the pathoplastic moderating effect of education on the association between depressive mood and SRH among U.S. cancer survivors using a national representative dataset. In addition to confirming the robust literature on social determinants of health articulating the negative impact of depressive mood and the protective impact of education on cancer survivors’ SRH [31,32], this study reveals that the negative association between depressive mood and SRH is significantly greater among U.S. cancer survivors with higher levels of education, i.e., bachelor’s degree or higher, when compared to their counterparts with below high school education.

Given the well-established protective role of educational attainment for SRH, it is reasonable to anticipate that, for cancer survivors, the negative impact of depressive mood on SRH may become weaker among those with a higher level of education, i.e., education buffers the harmful effect of depressive mood on SRH. However, the findings of this study reveal a converse relationship in that the negative association between depressive mood and SRH is significantly stronger among those with higher levels of education. Thus, even though higher education is a protective factor of SRH among cancer survivors, SRH among those with higher education was found to be more vulnerable to depressive mood than those with lower education.

As indicated earlier in the introduction, education’s dual role as a “protective-risk” factor for SRH among cancer survivors is pathoplastic in nature [18]. While cancer survivors’ educational attainment, in general, protects individuals from depression and improves their SRH, individuals with high levels of education are more likely to experience severe and recurrent episodes of major depression than their counterparts with low levels of education. Thus, for cancer survivors who are depressed, those with high levels of education are likely to manifest more severe depressive symptomologies than their peers with low levels of education. The specific mechanism articulating how educational attainment impacts cancer survivors’ personalities and, consequently, interacts with their depressive disorders is beyond the scope of this paper and should be further evaluated. The findings of this study articulate the importance of accounting for pathoplasticity combined with the social determinants of health frameworks, such as education, when evaluating the relationship between depressive mood and SRH among cancer survivors.

In addition to the pathoplastic influence of education on cancer survivors’ SRH, the findings of this study may be explained by the varied coping behaviors among cancer survivors with different educational backgrounds. For example, studies have reported that individuals with a higher level of education are more likely to use rumination and self-criticism to cope with depression [33]. Cancer survivors who continue to face a sequela of side- and late-effects due to cancer treatment tend to experience a greater risk of worsening mood when ruminating on their negative emotions and perceived poor health, which is often caused by cancer treatment-related symptoms such as pain, fatigue, or insomnia. As a result, cancer survivors who often use rumination and self-criticism to cope with depression, i.e., those with higher educational attainment, are more likely to have worse depression and lower self-rated health.

Several limitations should be noted. First, this study is cross-sectional and cannot infer causality, limiting the internal validity of study findings. This highlights the need for future longitudinal and national representative studies of cancer survivors to confirm these findings. Second, this study only included participants representative of U.S. adult cancer survivors who are 18 years or older; thus, findings are only generalizable to adult cancer survivors. Separate investigations focusing on pediatric, adolescent, and young adult cancer survivors are warranted to determine if these findings are replicable for younger cancer survivors. Third, cancer survivors’ depressive mood was evaluated using PHQ-8. Although with high validity and reliability, PHQ-8 does not provide diagnostic information of an individual’s clinical depression, another limitation of this present investigation. Finally, the dataset does not contain variables that indicate a cancer survivor’s current treatment stage, history of depression, or chronicity of current depression, all of which are important clinical covariates that should be accounted for when these variables become available.

Despite these limitations, the results of this study have important clinical implications for psycho-oncology providers. When working with cancer survivors who experience depression and low SRH, the assessment of social determinants of health remains an essential component in understanding cancer survivors’ general health status in order to ensure cancer survivors’ quality of life. More importantly, psycho-oncologists should extend beyond the direct protective impact of education on SRH among cancer survivors, especially given the pathoplastic moderating role of education on the relationship between depressive mood and SRH. Differential consideration is warranted for the role of educational attainment among cancer survivors with or without a depression diagnosis. While it is reasonable to view education as a protective factor for cancer survivors with cancer, psycho-oncology providers should pay particular attention to the symptom profile of depression for depressed cancer survivors with high levels of education.

For oncology providers supporting the general health and mental health wellness of individuals diagnosed with cancer, it is important to (1) account for the pathoplastic impact of education on cancer survivors’ depressive mood and self-rated health, (2) evaluate the depressive symptom profile for individuals diagnosed with cancer, and (3) evaluate specific coping styles of an individual patient diagnosed with cancer to inform the optimum selection and delivery of health and mental health support services [34,35,36].

## 5. Conclusions

Consistent with the social determinants of health literature, this study finds that depression and education correlate significantly with cancer survivors’ SRH. While education is positively associated with cancer survivors’ SRH, based on the theoretical framework of pathoplasticity, the negative association between depression and SRH is significantly stronger among those with high levels of education. Such a finding makes education a pathoplastic factor, i.e., a protective risk factor, for cancer survivors’ SRH, especially those who experience depression. Future studies are encouraged to evaluate the specific mechanism of such a relationship further and to develop evidence-informed practice guidelines for psycho-oncologists to improve cancer survivors’ self-rated health.

## Figures and Tables

**Table 1:** Participants’ Characteristics (*N* = 3844).

Variables	Mean (SD)		Percentage	*N* (Missing)
Self-Rated Health				3
Poor			8.59%	
Fair			18.51%	
Good			32.10%	
Very good/excellent			40.80%	
Depression	11.019	4.337		55
Education				26
Below high school			8.56%	
High school or equivalent			24.25%	
Some college			30.41%	
Bachelor’s degree			21.37%	
Above bachelor’s degree			15.40%	
Age Group				
18–39 years old			4.6%	
40–64 years old			31.14%	
65 years old and above			64.26%	
Sex				0
Male			41.57%	
Female			58.43%	
Race				0
Hispanic			5.05%	
NH White			86.08%	
NH Black/African American Only			5.52%	
NH Asian Only			1.22%	
NH American Indian/Alaska Native Only			0.57%	
NH American Indian/Alaska Native and Other Races			1.04%	
Other Single or Multiple Races			0.52%	
Income				0
Below 100% FPL			8.74%	
100%–below 200% FPL			18.05%	
200%–below 300% FPL			17.27%	
300%–below 400% FPL			13.27%	
400%–below 500% FPL			10.85%	
500% FPL and above			31.82%	
Marital Status				82
Married			49.55%	
Separated			1.04%	
Divorced			18.85%	
Single/never married			8.96%	
Widowed			21.61%	
Residential Setting				0
Large central metro area			22.71%	
Large fringe metro area			24.74%	
Medium and small metro area			33.84%	
Nonmetropolitan			18.70%	
Physical Health Conditions				0
Having hypertension			55.87%	
Having high cholesterol			46.87%	
Having asthma			10.61%	
Having diabetes			15.58%	
Having COPD, emphysema, or chronic bronchitis			11.65%	
Having arthritis			46.72%	
Having coronary heart disease			11.70%	
Cancer diagnosis ^†^			--	0

Note: Categorical variables were summarized using percentages. Continuous variables were summarized using mean and standard deviation (SD). Descriptive statistics for the 29 types of cancers were omitted in the table. ^†^ Descriptive statistics for cancer diagnosis are not presented for parsimonious reasons; included cancer diagnoses are: bladder cancer; blood cancer (leukemia, lymphoma, myeloma); bone cancer; brain cancer; breast cancer; cervical cancer; colon cancer; esophageal cancer; gallbladder cancer; larynx–trachea cancer; liver cancer; lung cancer; lymphoma cancer; melanoma; mouth, tongue, or lip cancer; ovarian cancer; pancreatic cancer; prostate cancer; rectal cancer; skin melanoma cancer; skin non-melanoma cancer; skin cancer (do not know what kind); stomach cancer; throat cancer; thyroid cancer; uterine cancer; head and neck cancer; colorectal cancer; other cancer.

**Table 2:** Association between Depression, Education, and Self-Rated Health (*N* = 3844).

Variables	Model 1 ^a^				Model 2 ^b^			
	OR ^c^		95% CI		OR		95% CI	
Depression	0.863	*** ^d^	0.840–0.886		0.909	***	0.862–0.959	
Education (ref: below high school)								
High school or equivalent	1.476	*	1.081–2.015		3.185	**	1.380–7.354	
Some college	1.762	***	1.302–2.384		2.804	**	1.303–6.035	
Bachelor’s degree	2.358	***	1.692–3.285		7.836	***	3.529–17.400	
Above bachelor’s degree	2.039	***	1.440–2.888		10.880	***	3.950–29.967	
Depression × Education								
Depression × high school or equivalent					0.938		0.872–1.010	
Depression × some college					0.965		0.906–1.027	
Depression × bachelor’s degree					0.900	**	0.837–0.967	
Depression × above bachelor’s degree					0.859	***	0.785–0.941	
Age Group (ref: 18–39 years old)								
40–64 years old	0.787		0.472–1.313		0.821		0.494–1.363	
65 years old and above	0.862		0.512–1.452		0.885		0.527–1.488	
Female (ref: male)	1.489	***	1.179–1.881		1.492	***	1.182–1.884	
Race (ref: Non-Hispanic (NH) White)								
Hispanic	0.463	***	0.318–0.675		0.467	***	0.323–0.676	
NH Black/African American Only	0.760		0.544–1.062		0.755		0.543–1.051	
NH Asian Only	0.488		0.231–1.028		0.487		0.227–1.047	
NH American Indian/Alaska Native Only	0.729		0.195–2.721		0.723		0.202–2.585	
NH American Indian/Alaska Native and Other Races	0.722		0.296–1.757		0.676		0.270–1.688	
Other Single or Multiple Races	1.129		0.472–2.701		1.064		0.434–2.612	
Income (ref: below 100% FPL)								
100%–below 200% FPL	2.176	***	1.467–3.226		2.220	***	1.505–3.277	
200%–below 300% FPL	2.177	***	1.501–3.158		2.276	***	1.578–3.283	
300%–below 400% FPL	2.833	***	1.896–4.232		2.910	***	1.955–4.331	
400%–below 500% FPL	2.756	***	1.751–4.339		2.813	***	1.803–4.389	
500% FPL and above	3.657	***	2.478–5.399		3.666	***	2.491–5.394	
Marital Status (ref: married)								
Separated	0.957		0.458–2.003		0.968		0.456–2.052	
Divorced	1.012		0.796–1.286		1.015		0.798–1.292	
Single/never married	0.945		0.649–1.375		0.966		0.664–1.404	
Widowed	1.057		0.844–1.325		1.060		0.845–1.329	
Residential Setting (ref: urban, large central metro area)								
Large fringe metro area	1.184		0.935–1.499		1.177		0.929–1.490	
Medium and small metro area	1.023		0.815–1.284		1.010		0.806–1.267	
Non-metropolitan area	0.898		0.704–1.144		0.892		0.700–1.136	
Having hypertension	0.559	***	0.473–0.659		0.558	***	0.473–0.659	
Having high cholesterol	0.919		0.778–1.085		0.921		0.779–1.088	
Having asthma	0.717	*	0.543–0.948		0.709	*	0.537–0.938	
Having diabetes	0.692	***	0.556–0.863		0.694	***	0.557–0.864	
Having COPD, emphysema, or chronic bronchitis	0.373	***	0.279–0.498		0.369	***	0.277–0.492	
Having arthritis	0.644	***	0.548–0.758		0.647	***	0.550–0.763	
Having coronary heart disease	0.506	***	0.384–0.668		0.496	***	0.377–0.653	

Note: ^a^ Model 1 is the main effect model. ^b^ Model 2 is the interaction effect model. ^c^ All coefficients were estimated after adjusting for sample weights and types of cancers. ^d^ * *p* < 0.05, ** *p* < 0.01, *** *p* < 0.001.

**Table 3:** Association between Depression and Self-Rated Health by Education among Older Cancer Survivors (*N* = 2470).

Education	Association between Depression and Self-Rated Health		
	OR ^a^		95% CI ^a^
Below high school	0.909	*** ^b^	0.862–0.959
High school or equivalent	0.853	***	0.810–0.898
Some college	0.877	***	0.845–0.910
Bachelor’s degree	0.818	***	0.775–0.864
Above bachelor’s degree	0.781	***	0.725–0.843

Note: ^a^ Both odds ratios (ORs) and 95% confidence intervals were estimated after adjusting for sample weights and covariates. ^b^ ** *p* < 0.01; *** *p* < 0.001.

## Data Availability

Dataset is available at: https://www.cdc.gov/nchs/nhis/data-questionnaires-documentation.htm.
